# The role and performance of chest X-ray for the diagnosis of tuberculosis: A cost-effectiveness analysis in Nairobi, Kenya

**DOI:** 10.1186/1471-2334-5-111

**Published:** 2005-12-12

**Authors:** MRA van Cleeff, LE Kivihya-Ndugga, H Meme, JA Odhiambo, PR Klatser

**Affiliations:** 1 KNCV Tuberculosis Foundation, The Hague, The Netherlands; 2 Centre for Respiratory Diseases Research (CRDR), Kenya Medical Research Institute (KEMRI), Nairobi, Kenya; 3 Centre for Diseases Control (CDC) Nairobi, Kenya; 4 KIT Biomedical Research, KIT (*Koninklijk Instituut voor de Tropen/Royal Tropical Institute*) Amsterdam, The Netherlands

## Abstract

**Background:**

The objective of this study was to establish 1) the performance of chest X-ray (CXR) in all suspects of tuberculosis (TB), as well as smear-negative TB suspects and 2) to compare the cost-effectiveness of the routine diagnostic pathway using Ziehl-Neelsen (ZN) sputum microscopy followed by CXR if case of negative sputum result (ZN followed by CXR) with an alternative pathway using CXR as a screening tool (CXR followed by ZN).

**Methods:**

From TB suspects attending a chest clinic in Nairobi, Kenya, three sputum specimens were examined for ZN and culture (Lowenstein Jensen). Culture was used as gold standard. From each suspect a CXR was made using a four point scoring system: i: no pathology, ii: pathology not consistent for TB, iii: pathology consistent for TB and iv: pathology highly consistent for TB. The combined score i + ii was labeled as "*no TB*" and the combined score iii + iv was labeled as "*TB*". Films were re-read by a reference radiologist. HIV test was performed on those who consented. Laboratory and CXR costs were used to compare for cost-effectiveness.

**Results:**

Of the 1,389 suspects enrolled, for 998 (72%) data on smear, culture and CXR was complete. 714 films were re-read, showing a 89% agreement (kappa value = 0.75 s.e.0.037) for the combined scores "*TB*" or "*no-TB*". The sensitivity/specificity of the CXR score *"TB" *among smear-negative suspects was 80%/67%. Using chest CXR as a screening tool in all suspects, sensitivity/specificity of the score "*any pathology*" was 92%, respectively 63%. The cost per correctly diagnosed case was for the routine process $8.72, compared to $9.27 using CXR as screening tool. When costs of treatment were included, CXR followed by ZN became more cost-effective.

**Conclusion:**

The diagnostic pathway ZN followed by CXR was more cost-effective as compared to CXR followed by ZN. When cost of treatment was also considered CXR followed by ZN became more cost-effective. The low specificity of chest X-ray remains a subject of concern. Depending whether CXR was performed on all suspects or on smear-negative suspects only, 22%–45% of patients labeled as "TB" had a negative culture. The introduction of a well-defined scoring system, clinical conferences and a system of CXR quality control can contribute to improved diagnostic performance.

## Background

Since the World Health Organization (WHO) introduced the DOTS strategy in 1993 for the control of tuberculosis (TB), Chest X-ray (CXR) has been discouraged for the diagnosis of TB [1]. As TB is mainly transmitted by sputum smear-positive patients, the DOTS strategy strongly promotes smear microscopy for the diagnosis of TB among symptomatic patients, the so-called TB suspects. Chest X-ray is restricted to diagnosing smear-negative TB among those suspects whose sputum examination is negative [2]. Smear microscopy with Ziehl-Neelsen (ZN) staining is mostly used. Because of its low specificity, if diagnosis among TB suspects would be based on CXR, this would lead to a substantial proportion (37%) of over-diagnosis [3]. But even, when restricting CXR for the diagnosis of smear-negative TB among smear-negative suspects, the proportion of over-diagnosis remains high (23%) [4].

The performance of CXR expressed as sensitivity and specificity to pick-up culture-positive TB cases depends on the intensity and the presentation of the disease, which in turn is influenced by a range of other factors. A major factor is the HIV status of the patient. In mild immunocompromised TB patients, the appearance of the CXR is often classical with cavitations and upper lobe infiltrates, while in severe immunocompromised TB patients, the appearance is often atypical [5]. Other factors influencing the presentation of the disease on the CXR film are delay in diagnosis and the sex of the patient [6]. Moreover, these factors are also interdependent of each other [5,6].

Another important factor is the experience and the interpretation skill of the reader [3], making CXR subject to intra- and inter-reader variation. Studies conducted in the 1950s showed that readers have a tendency to under-read (21 – 39%) rather than to over-read (2–19%) [3], with less discrepancy when readers were more experienced. A study in Japan using Miniature Mass Radiography found that around 20% of the cases with active TB were missed [3]. The well- known IUATLD study on X-ray classification in which 1,100 films were read by 90 experienced physicians and radiologists from 9 countries, found up to 34% disagreement on the question: "is the film normal?" and a 28% disagreement on the question: "is there a cavity present?" [3,7,8]. Finally, the performance of CXR also depends on the quality of the film, which depends on the functioning of the CXR machine, the reagents and the developing process. In addition to the fact that CXR is unable to distinguish 'smear-positive TB' from 'smear-negative TB', all above-mentioned factors contribute to certain degrees of over- and under-diagnosis.

Three steps are recommended as part of the diagnostic process and is widely practiced in most sub-Saharan countries. Step one is the identification of TB suspects among clinic attendees. Step two is the delivery of three sputa for smear microscopy for the diagnosis of 'smear-positive TB'. When all three smear results are negative, the TB suspects enter step three for a CXR for the diagnoses of 'smear-negative TB'. Although some patients may first start a course of broad-spectrum antibiotics before entering step three, the role of CXR for the diagnosis of 'smear-negative TB' is paramount.

Due to the large number of TB suspects that needs to be examined by smear microscopy to detect a TB patient, as is the case in many sub-Saharan cities, adherence to the prescribed diagnostic procedures is often difficult. This counts for both the laboratory technicians as well as for the patients. When these procedures are not strictly followed, misclassification and under-diagnosis may occur [4]. In such settings, an alternative diagnostic pathway can be used, in which all TB suspects are first subjected to a CXR leaving smear microscopy only for those suspects showing pathology on the CXR (CXR followed by ZN).

**Figure 1:**
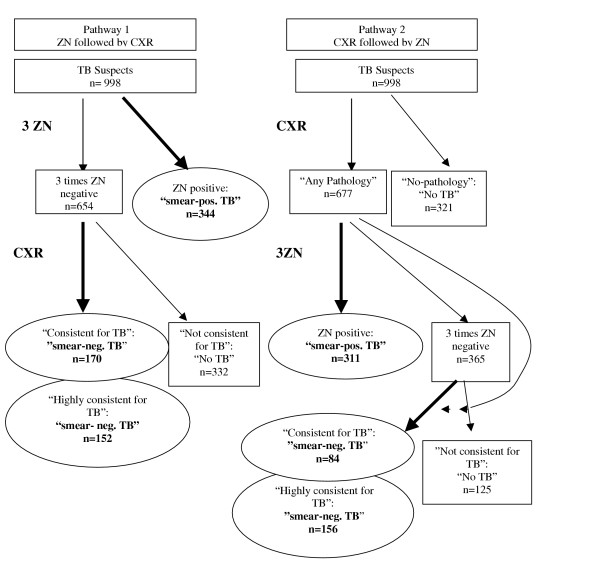
Flowchart of the two diagnostic pathways.

In this study, we calculated the performance of CXR in two different patient groups (all TB suspects and the smear-negative suspects. We also studied the cost-effectiveness of the two diagnostic pathways; 1) the routine diagnostic pathway of smear microscopy followed by CXR on those suspects with negative smear results (ZN followed by CXR) and 2) the alternative pathway using CXR as a screening tool by subjecting only those suspects to a ZN smear who showed any form of pathology on the CXR (CXR followed by ZN) (Figure 1).

## Methods

Between March 2000 and March 2001 TB suspects, aged 15 to 65 years, attending Rhodes Chest Clinic (RCC) in Nairobi were enrolled into the study. A TB suspect was defined as somebody presenting at the clinic with a cough of more than 3 weeks and/or symptoms of haemoptysis.

TB suspects were counseled to obtain informed consent and to deliver three sputum samples. A spot specimen was collected at the first attendance day, an early morning sputum was collected the next day at home and a third spot specimen collected when the patient brought his/her morning sputum to the clinic. Each sputum specimen was examined using ZN smear microscopy and Mycobacterial culture [3]. A slide was labelled ZN positive if one acid fast bacillus was seen in reading a minimum of at least 100 fields. For culture a volume of 0.25 ml of each decontaminated processed sputum specimen was inoculated onto slants of Lowenstein-Jensen (LJ) medium in culture bottles (Becton Dickenson Microbiology Systems, Cockeysville, MD, USA). The cultures were incubated at 37°C for 8 weeks and examined for growth twice weekly for the first 2 weeks and weekly thereafter until a definitive result was obtained [9,10]. Accuprobe^® ^(GenProbe, San Diego, CA, USA) testing identified 98.7% of all culture-positive samples as *M. tuberculosis *and a small proportion (1.3%) was identified as mycobacterium other than *M. tuberculosis *(MOTT)[10].

All TB suspects had a CXR. As part of the routine diagnostic procedures of the Kenyan National Leprosy and Tuberculosis Programme [11], initially CXR was performed only on those suspects whose three sputum smear results were ZN negative. From those who had a positive ZN smear result a CXR was also taken, however after the diagnosis was made. A four point scoring system was introduced to report on the CXR results (i: no pathology, ii: pathology not consistent for TB, iii: pathology consistent for TB and iv: pathology highly consistent for TB). A patient was labeled as 'TB" when the CXR showed pathology consistent and/or highly consistent for TB (CXR score iii and/or iv). Features such as solitary hilar and mediastinal shadows, and diffuse small nodular shadows or pleural effusion were considered as consistent for TB. Patchy or nodular shadows, cavitations and calcified shadows were considered as highly consistent with TB. The radiologist of RCC established the score. A random selection was re-scored by a reference radiologist, blinded for the outcomes of the RCC reader and the ZN smear results.

According to the Kenyan guidelines [11], a patient was labeled having 'smear-positive TB' if at least one out of three sputum examination results was ZN positive. (This is in contrast to other countries where at least two sputum examinations should be positive). A patient was defined as 'smear-negative TB' when three sputum smears were ZN negative and the CXR scored '*TB*'. Culture was used as gold standard. A culture-positive patient having at least one positive culture result out of three interpretable results was regarded as a proven TB case. A patient with three negative culture results was regarded as a non-TB case.

Patients were also counseled for HIV testing on a voluntary basis Antibodies to HIV were determined by the Virinostika HIV Uni-Form II *plus 0 *assay from Organon Teknika (Boxtel, The Netherlands). Those who did not want an HIV test remained eligible for study inclusion. For this study a patient was scored HIV positive, if one test was positive.

Direct costs were established as described earlier and concerned costs to screen 998 suspects [12]. Labor costs were calculated from salary scales and routine allowances of the staff involved. The costs of materials and equipment for routine screening were included. Only laboratory costs and cost to establish a CXR were used to compare the cost-effectiveness of the two diagnostic processes. Cost analysis was based on processing a maximum of 50 ZN slides and 50 CXRs per day, which was an average performance of the clinic. Two trained staff performed each procedure. To follow a full course of treatment only health service costs were included. Cost-effectiveness analysis was used to compare the two diagnostic pathways. A correctly diagnosed case, defined as a culture-positive patient, was used as the effect. A sensitivity analysis was made to assess to what extent change in different TB prevalence environments would affect cost-effectiveness. Costs are expressed in US$, using an exchange rate of US$ 1 = 74 KSh (Kenyan Shillings)

Data were analyzed using Epi info and SPPS statistical software. Chi-square test was used to compare binary data. Likelihood ratios were used for the sensitivity analysis. Logistic regression was performed on culture positive TB patients to assess the impact of HIV on test performance.

## Results

In total 1,389 suspects were enrolled. A result on all three sputum samples and a CXR was available for 998 (72%) suspects, forming the study group. Of the remaining 391 (28%) suspects, 169 (12%) missed a third culture result, mainly due to contamination and 222 (16%) had no CXR taken, mainly because they did not return to the clinic. Characteristics of the patients not included were similar to the study group.

Of the study group, 559 (59%) were culture-positive, 600 (60%) were men and 398 (40%) women with a median age of 30 years and respectively 27 years (p =< 0.001). For 341 (34%) of the study group a sputum was positive for ZN, leaving 657 (66%) initially subjected to CXR for the diagnosis of smear-negative TB. In total, 714 (72%) CXRs were re-read. When comparing all four scores, the two readers showed an agreement of 70% (kappa value 0.55, s.e. 0.023), while for the combined 2-scale score "*TB*" or "*no-TB*" the agreement was 89% (kappa value = 0.75, s.e.0.037)

The proportion of men who consented to voluntary HIV testing was 29% as compared to 24 % of the women (p > 0.05). The seroprevalence was significantly higher among women than that among men (55.9% versus 33.9%, p = 0.0002).

Among the sputum smear-negative suspects, 49% (323/657) had a CXR score "*TB*", of which 180 (56%) were culture positive (Table 1). Of the 226 culture positive suspects, 80% (180/226) had a CXR result "*TB*", leaving 20% (46/226) with a score "*no- TB*" not picked-up.

**Table 1:** Results of CXR examination stratified by culture in TB suspects excluding those who were smear-positive on ZN microscopy. (N = 657).

	Total (N = 657)	Culture positive (N = 226)	Culture negative (N = 431)
a) Highly consistent for TB	152 (100%)	109 (72%)	43 (28%)
b) Consistent for TB	171 (100%)	71 (42%)	100 (58%)
c) Pathology, but no TB	22 (100%)	6 (27%)	16 (73%)
d) No pathology	312 (100%)	40 (13%)	272 (87%)

" TB"*: (a+b)	*323 (100%)*	*180 (56%)*	143 (44%)
*" no-TB": (c+d)*	*334 (100%)*	*46 (14%)*	288 (86%)

* Smear-negative pulmonary TB

When applying CXR on all suspects, the yield in detecting culture positive suspects with the combined score "Any pathology" was 92% (515/559), leaving 8 % culture-positive suspects undetected (Table 2).

**Table 2:** Results of CXR examination stratified by culture in all TB suspects. (N = 998).

	Total (N = 998)	Culture positive (N = 559)	Culture negative (N = 439)
a) Highly consistent for TB	423 (100%)	379 (90%)	44 (10%)
b) Consistent for TB	229 (100%)	127 (55%)	102 (45%)
c) Pathology, but no TB	27 (100%)	9 (33%)	18 (67%)
d) No pathology	319 (100%)	44 (14%)	275 (86%)
*"any pathology"(a+b+c)*	*679* (100%)	*515 (76%)*	164 (24%)
*" no-TB": (c+d)*	*364*(100%)	*53 (15%)*	293 (85%)

Table 3 shows the sensitivity/specificity for each of the CXR scores in different suspect groups, as well as for the entire diagnostic processes. Using CXR as a screening tool on all suspects by using the score "*any pathology" *the sensitivity was 92%, slightly higher when the score *"TB" *was used (91%). Using CXR as a diagnostic tool on smear-negative suspects, the sensitivity of the score *"TB" *was 80%. The sensitivity of the score "Highly consistent for TB" (48%) was significantly lower as compared to the similar score on all suspects.

**Table 3:** CXR performance on all suspects and on suspects excluding those who were ZN-positive. Culture (patient based) used as gold standard (95% confidence interval)

Score of the reader	Sensitivity	Specificity	PPV	NPV
**CXR on all suspects (n = 998)**				
a) Highly consistent for TB	68 (64–72)	90 (87–93)	0.90 (0.87–0.93)	0.69 (0.65–0.72)
b) Consistent for TB	23 (19–26)	77 (73–80)	0.55 (0.49–0.62)	0.44 (0.40–0.47)
c) Pathology, but no TB	2 (0–3)	96 (94–98)	0.33 (0.16–0.51)	0.43 (0.40–0.46)
*"TB" (a+b)*	91 (88–93)	67 (62–71)	0.78 (0.74–0.81)	0.84 (0.81–.088)
*"any pathology" (a+b+c)*	92 (90–94)	63 (58–67)	0.76 (0.73–0.79)	0.86 (0.82–0.90)
				
**CXR on suspects excluding those who were ZN positive (n = 657)**				
a) Highly consistent for TB	48 (42–55)	90 (87–93)	0.72 (0.65–0.79)	0.77 (0.73–0.81)
b) Consistent for TB	31 (25–37)	77 (73–81)	0.42 (0.34–0.49)	0.68 (0.83–0.90)
c) Pathology but no TB	3 (1–5)	96 (95–98)	0.27 (0.9–0.46)	0.65 (0.62–0.69)
*"TB" (a+b)*	80 (74–85)	67 (62–71)	0.56 (0.50–0.61)	0.86 (0.83–0.90)
				
**The entire diagnostic process (n = 998)**				
ZN followed by CXR	93 (91–95)	62 (57–67)	0.76 (0.72–0.79)	0.87 (0.83–0.91)
CXR followed by ZN	89 (86–91)	97(84–90)	0.90 (0.87–0.92)	0.86 (0.83–0.89)

The sensitivity of the routine diagnostic pathway (ZN followed by CXR) was 4% higher (93%) than the alternative pathway (89%) (CXR followed by ZN), leaving 7% and 11% respectively culture-positive cases undetected. As compared to the alternative pathway, the routine pathway was more sensitive for both for smear-positive, as well as for smear-negative TB cases.

Logistic regression was performed to identify whether age, sex and HIV were risk factors influencing the performances of CXR. Performing CXR on all suspects, the odds of having a CXR score "*TB*" was lower for women (aOR = 0.66, 95% CI 0.51–0.84, p < 0.01) than men, but there was no association with age or HIV. Restricted to smear-negative suspects, the result "*TB*" was associated with HIV, though this was not statistically significant (aOR 1.72, 95% CI 0.72–3.05, p = 0.064). The sensitivity of the score "Highly consistent for TB" among HIV- negative suspects was higher (77%) as compared to HIV-positive suspects (49%),

As earlier described [6], the sensitivity of the score "*TB*" among smear-negative suspects was higher for men (82%) than for women (77%). The specificity was more or less similar (66% versus 68%), and improved considerably when using the score "highly consistent with TB" (88% and 93%, respectively). Culture-positive men harbor more cavities than women (62% versus 50%) and also the average number of cavities among men (2.0) was significantly higher (p = 0.032) as compared to women (1.6).

Table 4 shows results of the two diagnostic pathways including the costs and cost-effectiveness to examine 998 TB suspects. The routine process detected 335 smear-positive TB cases, 9% more as compared to the alternative process. The cost-effectiveness per correctly diagnosed case for the routine process was slightly better (US$8.72 and US$9.27) however, when treatment costs were considered, including costs of treatment of those falsely diagnosed, the alternative pathway was more cost effective (US$ 137 versus US$ 158).

**Table 4:** Results of the two diagnostic pathways including costs* and cost-effectiveness in US$. (N = 998)

	ZN followed by CXR (n = 998).	CXR followed by ZN (n = 998)
Number of culture positive suspects	559	559
Number of patient put on treatment	666	551
Number Culture pos. patient put on treatment	514	496
*C+/ZN+ patients put on treatment*	*335*	*308*
*C+/ZN- patient put on treatment*	*179*	*188*
Number Culture neg. patients put on treatment (Over diagnosis)	152	56
Number Culture pos. patients not put on treatment (Under diagnosis)	45	63
Laboratory service		
Labour costs	$973	$973
Investment costs	$135	$135
Running costs	$1,706	$1,151
**Total laboratory costs**	**$2,814**	**$2,259**
X-ray service		
Labour costs	154	$154
Investment costs	237	$237
Running costs	1,278	$1,946
**Total CXR service costs**	**1,669**	**$2,337**
Total diagnostic costs	$4,483	$4,596
Cost per correctly diagnosed patient	$8.72	$9.27
		
Total treatment costs **	$76,565	$63,422
Diagnostic costs + treatment costs	$81,048	$68,018
Cost per correct diagnosed patient including costs of treatment.	$158	$137

* costs only include health service costs

** costs for health service to treat a patient: $115

**Figure 2:**
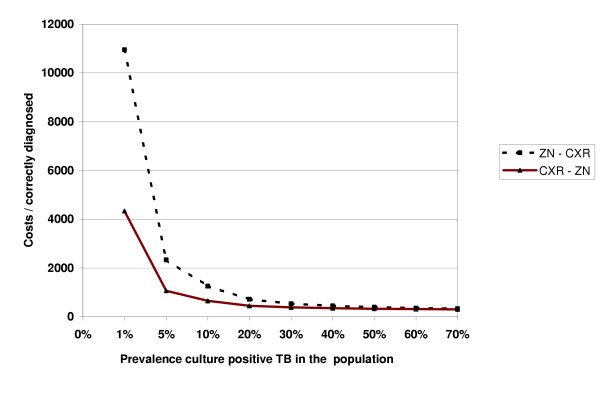
Cost effectiveness (including treatment costs) of two diagnostic processes (ZN followed by CXR and CXR followed by ZN) for different prevalence of culture positive TB in the suspect population.

Figure 2 shows a sensitivity analysis comparing the cost-effectiveness of the two diagnostic pathways, including costs of treatment for different proportions (prevalence) of culture-positive cases among all suspects. In settings with a low TB prevalence, CXR used as a screening tool was more cost-effective, while in settings with a TB prevalence of more then 40% the cost-effectiveness of both diagnostic processes was almost equal.

**Figure 3:**
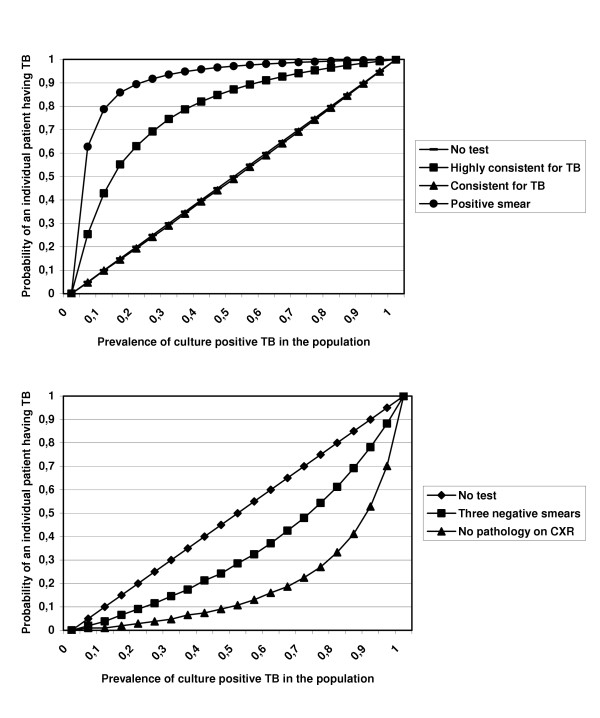
A: Predictive values of ZN microscopy and the CXR scores: "Highly consistent for TB" and "Consistent for TB" for having a positive culture result for *M. tuberculosis*. B: Predictive values of ZN microscopy (three negative results), CXR score "No pathology" to exclude culture-positive TB.

Figure 3a and 3b show the probability of a patient having TB using ZN microscopy and CXR in settings with different TB prevalence. A positive ZN smear result was the best test to predict a culture-positive case; while the CXR score '*No pathology*' was the best test to rule-out TB.

## Discussion

Many studies indicate that CXR is unreliable for the diagnosis of TB [3]. In contrast to other studies [3], we found little difference between the reader at RCC and the reference reader. Our study showed that the performance of CXR expressed as sensitivity and specificity in picking-up culture positive TB patients, differed among different patient groups. When cavities are present, which is more commonly seen in far-developed smear-positive TB, the interpretation of the film may be easier, resulting in a higher sensitivity and specificity. This clarifies why for example, the sensitivity of the score "Highly consistent for TB" in all suspects was 68%, while the same score was only 48% among the smear-negative suspects. Cavities are also less pronounced among HIV-positive TB cases, which clarifies why among these groups the sensitivity of CXR was reduced as well, an observation consistent with a study conducted in Spain where a considerable number of HIV positive TB patients had a normal CXR [13]. For similar reasons, the sensitivity among women was found lower as compared to men [6].

The presentation of TB, and consequently the performance of the CXR reading, is also influenced by delay in accessing diagnosis with longer delays associated with high numbers of cavities. Such findings were consistent with a study in Canada, showing over a 10 year period, an increase in normal CXRs from 1% to 10% among proven TB cases, which was associated with earlier diagnosis [14]

The low specificity of CXR remains a subject of concern. When the CXR was labeled as "*TB*", comprising the combined score "Highly consistent for TB" and "Consistent for TB", the specificity was low (67%). As a consequence, the number of patients labeled as having TB using CXR with a negative culture that were placed treatment was rather high: 22% among all suspects and 45% among smear-negative suspects.

The challenge is to increase the specificity of CXR and diminish the proportion of over-diagnosis of smear-negative cases. The introduction of the four point scoring system, using only the score "Highly consistent for TB" for diagnosing TB and starting a course of broad-spectrum antibiotics on those suspects with a score "Consistent for TB" may improve performance. By doing so, we found that over-diagnosis could be reduced up to 67%, while only 8% fewer culture positive cases would start immediate treatment.

Regarding diagnosis of smear-negative TB, the clinician often only relies on the CXR result. The radiologist in turn usually has little or no information about the patient and in case of doubt may tend to give a positive result. Moreover, although the interpretation of CXR is more complicated than that of smear examination, quality control is hardly practiced for CXR.

The global DOTS strategy advocates that diagnosis is based on smear microscopy and restricts CXR only for the detection of "smear-negative TB cases". Our study supports this strategy. The routine diagnostic process (ZN followed by CXR) detected 9% more culture and 9% more smear-positive TB cases as compared to the alternative process (CXR followed by ZN).

When dealing with many TB suspects, the alternative pathway using CXR as a screening tool is often considered as being cost-effective [15]. When only direct costs of laboratory and CXR were included, we found the routine diagnostic process is more cost effective. One should however take into account that the high numbers of patients over-diagnosed, and who are falsely put on treatment, also implies other costs. When treatment costs of all patients were included in the analysis, including the treatment of those culture-negative patients, the cost-effectiveness of the alternative process became slightly better. Emphasis to reduce the falsely diagnosed patients is therefore important.

The prevalence of culture-positive TB cases among the study group was rather high (59%) and may be biased by the proportion of suspects (16%) who did not return to the clinic for a CXR and were not included in the study population, and who all were culture-negative. In the sensitivity analysis (including treatment costs) adjusting for the prevalence of TB, the alternative pathway remained more cost-effective, but became more pronounced in settings with TB prevalence lower than 30%, as for example is the case in total population prevalence surveys.

In addition to diagnosis, CXR play also a role in HIV/TB combined programs. More countries are implementing Isoniazid preventive therapy for HIV infected persons to reduce the development of TB [16]. But before starting a course of Isoniazid one should rule out active TB. Although a study performed in Botswana suggested otherwise [17], we found that the CXR score "No pathology" with a certainty of over 90% rules out active TB.

## Conclusion

In our study, the routine diagnostic pathway (ZN followed by CXR) identified a higher number of smear-positive cases as compared to the alternative process (CXR followed by ZN) and was also more cost-effective. The low specificity of CXR remains a subject of concern. Depending on the group of patients, 22%–45% of cases with a CXR result "*TB*" and consequently put on treatment, had a negative culture.

From the findings of the study, we believe that the introduction of a clearly defined four point scoring system for CXR for the interpretation of CXR may well improve the diagnostic performance. Consequently those patients with a score "Highly consistent for TB" can start directly anti-TB treatment, while those patients with a score "Consistent for TB" can be considered for a course of broad spectrum antibiotics with appropriate follow-up.

Being a function of many factors, the performance of CXR could also improve when the radiologist, through clinical conferences, would know the underlying information of the patients, such as treatment history, age or HIV status. Moreover, being so important members of the diagnostic team, radiologists should be included in NTP training programs. Finally, until we have new diagnostic tests available for field use, we need to ensure not only the quality of smear microscopy, but also the quality of CXR. The latter could be improved through the introduction of quality systems.

## Competing interests

The author(s) declare that they have no competing interests.

## Authors' contributions

All authors participated equally in the design, coordination, analysis and writing of the study.

## Pre-publication history

The pre-publication history for this paper can be accessed here:


